# Quetiapine-induced sleep-related eating disorder-like behavior: a case series

**DOI:** 10.1186/1752-1947-6-380

**Published:** 2012-11-06

**Authors:** Sadeka Tamanna, M Iftekhar Ullah, Chelle R Pope, Garland Holloman, Christian A Koch

**Affiliations:** 1 Department of Sleep Medicine, G. V (Sonny) Montgomery VA Medical Center, Jackson, MS 39216, USA; 2 Division of General Internal Medicine, Department of Medicine, University of Mississippi Medical Center, Jackson, MS 39216, USA; 3 Division of Pulmonary, Critical Care and Sleep medicine, Department of Medicine, University of Mississippi Medical Center, Jackson, MS 39216, USA; 4 Department of Psychiatry, University of Mississippi Medical Center, Jackson, MS 39216, USA; 5 Department of Medicine, G. V (Sonny) Montgomery VA Medical Center, Jackson, MS 39216, USA; 6 Division of Endocrinology, Department of Medicine, University of Mississippi Medical Center, Jackson, MS 39216, USA; 7 Department of Medicine, University of Dresden, Dresden, Germany

**Keywords:** Quetiapine, SRED, Somnambulism, Sleep eating, Sleepwalking, Obesity

## Abstract

**Introduction:**

Somnambulism or sleepwalking is a disorder of arousal from non-rapid eye movement sleep. The prevalence of sleep-related eating disorder has been found to be approximately between 1% and 5% among adults. Many cases of medication-related somnambulism and sleep-related eating disorder-like behavior have been reported in the literature. Quetiapine, an atypical antipsychotic medication, has been associated with somnambulism but has not yet been reported to be associated with sleep-related eating disorder.

**Case presentation:**

Case 1 is a 51-year-old obese African American male veteran with a body mass index of 34.11kg/m^2^ and severe sleep apnea who has taken 150mg of quetiapine at bedtime for more than one year for depression. He developed sleepwalking three to four nights per week which resolved after stopping quetiapine while being compliant with bi-level positive pressure ventilation therapy. At one year follow-up, his body mass index was 32.57kg/m^2^.

Case 2 is a 50-year-old African American female veteran with a body mass index of 30.5kg/m^2^ and mild sleep apnea who has taken 200mg of quetiapine daily for more than one year for depression. She was witnessed to sleepwalk three nights per week which resolved after discontinuing quetiapine while being treated with continuous positive airway pressure. At three months follow-up, her body mass index was 29.1kg/m^2^.

**Conclusion:**

These cases illustrate that quetiapine may precipitate complex motor behavior including sleep-related eating disorder and somnambulism in susceptible patients. Atypical antipsychotics are commonly used in psychiatric and primary care practice, which means the population at risk of developing parasomnia may often go unrecognized. It is important to recognize this potential adverse effect of quetiapine and, to prevent injury and worsening obesity, discuss this with the patients who are prescribed these medications.

## Introduction

Sleep-related eating disorder (SRED) represents recurrent episodes of involuntary eating and drinking during the main sleep period with one or more features of consumption of peculiar foods, insomnia, sleep-related injury, dangerous behaviors performed while in pursuit of food, morning anorexia and adverse health consequences from recurrent binge eating of high caloric food [1-5]. Somnambulism or sleepwalking is a disorder of arousal from non-rapid eye movement (NREM) sleep which often co-exists with SRED [3,6].

Many cases of medication-related somnambulism and SRED-like behavior have been reported in the literature. Zolpidem is one of the most commonly reported medications associated with these conditions. Quetiapine (Seroquel), a piperazinyl-dibenzothiazepine analog of clozapine, is an atypical antipsychotic medication frequently used for the treatment of schizophrenia, bipolar disorder and major depressive disorder as an adjunct to an antidepressant. It has been infrequently reported to be associated with sleepwalking [7] and in one case, it has actually been used to treat sleepwalking [8]. However, to the best of our knowledge, quetiapine has not yet been reported to be associated with the development of SRED.

We here report two cases of SRED which we believe were induced or aggravated by quetiapine because in both cases the condition resolved completely after discontinuation of the medication. In addition, both patients lost weight with a declining body mass index (BMI).

## Case presentation

### Case 1

A 51-year-old African American male veteran presented to our sleep clinic, accompanied by his wife, with complaints of daytime sleepiness, sleepwalking and eating during sleep for more than a year. He reportedly walks and eats uncooked food from the refrigerator and misplaces things during sleep three to four nights per week. His wife saw him walking around inside the house dressed up in his best outfit in the middle of the night carrying the car key. The patient could not remember any of these events after awakening.

His past medical history included depression, hypertension and mood disorder. There was no previous history of seizure, childhood or family history of parasomnia or alcohol abuse. His medications included bupropion, quetiapine, lisinopril, hydrochlorothiazide and loratadine. He was diagnosed with major depression about eight years ago and received bupropion 150mg two times a day for treatment. He underwent a major financial crisis with job loss and had to move to his mother’s house. His symptoms of depression were not improving and quetiapine was added about six years ago to help improve his depression. The dose of quetiapine was gradually titrated up to 150mg at bedtime by his psychiatrist. It is not clear when his SRED first started because the patient used to live alone until one year ago when he got married. His physical examination was unremarkable except for an elevated BMI of 34.11kg/m^2^ and poorly controlled hypertension. An overnight polysomnography was performed which revealed severe sleep apnea (apnea hypopnea index (AHI) of 86/hour with an arousal index of 156/hour). No parasomnia or periodic limb movements were noted during polysomnography. During the overnight titration study, his sleep apnea responded well to bi-level positive pressure ventilation (BiPAP) with optimal resolution of his apnea and hypopneas and there were not many treatment emergent central apneas. He was sent home on nightly BiPAP therapy and was scheduled for a follow-up in three months.

On his follow-up visit, he reported good compliance with his BiPAP (99% compliance for >4 hours per night for >4 nights per week, verified by the memory card installed in the BiPAP machine). His daytime sleepiness improved significantly but his wife complained of increased frequency of sleepwalking and eating during sleep almost every other night after starting him on BiPAP. Because quetiapine has been known to be associated with sleepwalking, he was advised to discontinue it. He returned for a follow-up in three months and, surprisingly, he did not have any further incidents of either SRED or sleepwalking. There was no change in any other medication except discontinuation of his quetiapine during this interval. He lost 0.91kg (2lb) during this three months follow-up. He was also followed up after one year of stopping quetiapine and he reported restful sleep without any further event of sleepwalking or eating during sleep. His BMI was 32.57kg/m^2^ and he had lost 4.08kg (9lb). His serum quetiapine level had never been measured.

### Case 2

A 50-year-old African American obese (BMI=30.5kg/m^2^) female veteran with a history of hypertension, asthma, major depressive disorder, migraine and obstructive sleep apnea presented to the clinic for follow-up. Her medications included verapamil, lisinopril, hydrochlorothiazide, bupropion, venlafaxine, topiramate and quetiapine.

She complained of walking in her sleep and eating from the refrigerator while she was asleep. These incidents were observed and reported by her niece and brother who lived in the same house. She was seen to walk and sit at the kitchen table with her eyes closed, eat cereal and go back to bed. They also mentioned that she started her electric coffee pot, putting water in it and went to bed at 3 a.m. She was having these episodes two to three nights per week. She was also diagnosed with obstructive sleep apnea after a baseline sleep study a few years back which showed obstructive sleep apnea (AHI = 10/hour) that was worse during REM sleep (REM AHI = 58/hour). She was prescribed continuous positive airway pressure (CPAP) at 8cm after adequate titration study but she was having trouble keeping her mask in place due to her sleepwalking episodes during which she removed the mask.

Over the past four years she was being treated with quetiapine for depression. This medication was added as an adjunct therapy when other medications did not control her symptoms adequately. Her quetiapine dose was increased to 200mg daily about a year ago for increasing depression from work-related stress. She could not give a correct history when the sleepwalking exactly started but it has been witnessed for six to eight months. As her medication list shows, she was already on topiramate for her migraine headache which was continued after stopping quetiapine. Although topiramate has been shown to be effective in SRED in a small trial [2], it did not help in her SRED symptoms. We tapered and eventually stopped her quetiapine. After three months, she reported during her follow up visit that she no longer had episodes of sleepwalking or sleep eating. There was no change in any other medication except discontinuation of her quetiapine during this interval. She could now use her CPAP every night and felt rested after her sleep. Her BMI was 29.1kg/m^2^ and she had lost 3.18kg (7lb).

## Discussion

SRED is characterized by recurrent episodes of partial arousals with involuntary eating during the main sleeping period, usually within the first three hours of sleep. A strong association between somnambulism and SRED has been reported. Somnambulism has been primarily linked to NREM sleep instability, particularly an abnormality in regulation of slow wave sleep (SWS). It is commonly precipitated by other factors including sleep deprivation, presence of other primary sleep disorders (sleep apnea, periodic limb movement disorder) as well as medications that raise the threshold for arousals [3].

Our patients had a few important predisposing factors for parasomnia including increased work-related stress, depression and severe sleep apnea with high arousal index. If a patient with parasomnia has any other concomitant primary sleep disorder, the treatment is initially directed towards that aspect which often resolves the parasomnia. Our first patient had severe sleep apnea that was treated adequately, but he continued to have sleepwalking and SRED despite good compliance with treatment probably because his SWS had increased after using BiPAP. Our second patient was not able to be compliant with the CPAP initially until she stopped taking quetiapine.

Sleepwalking and SRED have been commonly reported after taking zolpidem as well as sodium oxybate [9,10]. However, one case report has proposed quetiapine as a potential treatment of somnambulism because it decreases brain delta activity [8]. The somnambulism phenomenon from quetiapine may be explained by the serotonin hypothesis of parasomnia [11]. Quetiapine is found to block 5-hydroxytryptamine-2A (5HT-2A) and dopamine receptors subtype 2 (D2) increasing cortical dopamine release by 5HT-1A agonism [12]. The serotonergic neurons of the dorsal raphe nucleus (DRN) in the brain stem constitute an integral component for generation of SWS. Among a variety of subtypes of serotonergic receptors, 5HT-2A receptors in the DRN are known to regulate frequency and amplitude of SWS [13]. These neurons projecting into the ventrolateral preoptic area help maintain and increase the SWS. These serotonergic neurons are also thought to modulate the motor system by dampening the sensory input and attenuating cortical activation, which helps maintain the hypotonia of the antigravity muscles during SWS. Normally, maintenance of SWS is well coordinated with motor inhibition so that motor activity does not happen without an arousal. A blockade of serotonergic input can withdraw this motor inhibition, enabling the person to walk and perform other motor activities. Quetiapine has been known to alter central serotonin activity by blocking the 5-HT serotonergic receptor which may, in turn, dissociate these two components (state of sleep and muscle hypotonia) [12], leading to sleepwalking without a complete arousal.

**Figure 1:**
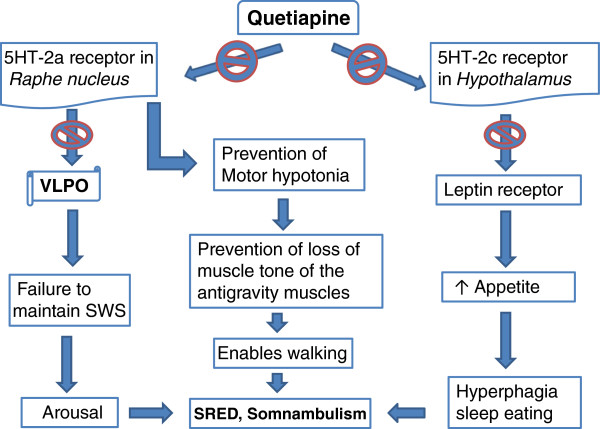
**Potential mechanism of quetiapine-induced SRED and somnambulism.** SRED: sleep-related eating disorder; SWS: slow wave sleep; VLPO: ventrolateral preoptic nucleus.

From the literature support described above, we propose a potential mechanism of quetiapine-induced SRED and somnambulism phenomenon explained in Figure 1. Sleepwalking aggravated by quetiapine may be explained by the above hypothesis; however, how it may also lead to eating during sleep is unclear. This may be again related to its effect on another serotonin receptor: 5HT-2C. The serotonin receptor 5HT-2C in the hypothalamus regulates mood, anxiety, feeding, and reproductive behavior [14]. Antagonism of the serotonin receptor 5HT-2C by antipsychotic drugs, including quetiapine, olanzapine and clozapine, increases appetite leading to increased food intake and weight gain [15]. Patients who are on these antipsychotic medications and gaining weight were also found to have elevated leptin levels [16]. Because quetiapine blocks 5HT-2C, it may cause leptin resistance at the level of the hypothalamus, contributing to increased food intake and obesity (Figure 1). Often, an obese body shape is not even perceived to be a problem or stressful, as assessed by the perceived stress scale [17]. 

## Conclusion

Quetiapine may increase the potential of SRED-like complex motor behavior in susceptible patients. SRED is more common than is generally recognized and the prevalence of SRED is high in the adult psychiatric population [18]. Atypical antipsychotics are commonly used in psychiatric and primary care practice, which puts the population at risk of getting parasomnia that may often go unrecognized. It is important to discuss this potential adverse effect with patients who are on these medications to prevent injuries and worsening obesity with its complications [19]. Further research is necessary to explore the exact mechanisms of how quetiapine can cause and/or aggravate SRED.

## Consent

Written informed consents were obtained from the patients for publication of this case series. Copies of the written consents are available for review by the Editor-in-Chief of this journal.

## Competing interests

The authors declare that they have no competing interests.

## Authors’ contributions

ST and CRP were part of the managing team and participated in collecting medical data during follow-up of the patients. ST and MIU were involved in the concept and design of the report and were major contributors in writing the manuscript. CAK and GH contributed to writing the manuscript. All authors were involved in, and contributed equally to, drafting the manuscript and approved the final manuscript.

## References

[B1] American Academy of Sleep Medicine International Classification of Sleep Disorders (ICSD-2): Diagnostic and Coding Manual 2005 2 Westchester: American Academy of Sleep Medicine

[B2] Winkelman JW Sleep-related eating disorder and night eating syndrome: sleep disorders, eating disorders, or both? Sleep 2006 29 876 877 16895252 10.1093/sleep/29.7.876

[B3] Schenck CH Hurwitz TD Bundlie SR Mahowald MW Sleep-related eating disorders: polysomnographic correlates of a heterogeneous syndrome distinct from daytime eating disorders Sleep 1991 14 419 431 1759095 10.1093/sleep/14.5.419

[B4] Morgenthaler TI Silber MH Amnestic sleep-related eating disorder associated with zolpidem Sleep Med 2002 3 323 327 10.1016/S1389-9457(02)00007-2 14592194

[B5] Allison KC Lundgren JD O'Reardon JP Geliebter A Gluck ME Vinai P Mitchell JE Schenck CH Howell MJ Crow SJ Proposed diagnostic criteria for night eating syndrome Int J Eat Disord 2010 43 241 247 19378289 10.1002/eat.20693 PMC4531092

[B6] Winkelman JW Clinical and polysomnographic features of sleep-related eating disorder J Clin Psychiatry 1998 59 14 19 10.4088/JCP.v59n0104 9491060

[B7] Hafeez ZH Kalinowski CM Somnambulism induced by quetiapine: two case reports and a review of the literature CNS Spectr 2007 12 910 912 18163036 10.1017/s1092852900015698

[B8] Gill JS Pillai SK Koh OH Jambunathan ST Low dose quetiapine in the treatment of an adolescent with somnambulism: a case report Acta Neurol Belg 2011 111 155 156 21748939

[B9] Guilleminault C Kirisoglu C da Rosa AC Lopes C Chan A Sleepwalking, a disorder of NREM sleep instability Sleep Med 2006 7 163 170 10.1016/j.sleep.2005.12.006 16459139

[B10] Wallace DM Maze T Shafazand S Sodium oxybate-induced sleep driving and sleep-related eating disorder J Clin Sleep Med 2011 7 310 311 21677903 10.5664/JCSM.1082 PMC3113972

[B11] Chiang A Krystal A Report of two cases where sleep related eating behavior occurred with the extended-release formulation but not the immediate-release formulation of a sedative-hypnotic agent J Clin Sleep Med 2008 4 155 156 18468314 PMC2335395

[B12] Seeman M Sleepwalking, a possible side effect of antipsychotic medication Psychiatr Q 2011 82 59 67 10.1007/s11126-010-9149-8 20734137

[B13] Ichikawa J Li Z Dai J Meltzer HY Atypical antipsychotic drugs, quetiapine, iloperidone, and melperone, preferentially increase dopamine and acetylcholine release in rat medial prefrontal cortex: role of 5-HT1A receptor agonism Brain Res 2002 956 349 357 10.1016/S0006-8993(02)03570-9 12445705

[B14] Puig MV Watakabe A Ushimaru M Yamamori T Kawaguchi Y Serotonin modulates fast-spiking interneuron and synchronous activity in the rat prefrontal cortex through 5-HT1A and 5-HT2A receptors J Neurosci 2010 30 2211 2222 10.1523/JNEUROSCI.3335-09.2010 20147548 PMC6634052

[B15] Heisler LK Zhou L Bajwa P Hsu J Tecott LH Serotonin 5-HT(2C) receptors regulate anxiety-like behavior Genes Brain Behav 2007 6 491 496 10.1111/j.1601-183X.2007.00316.x 17451451

[B16] Nonogaki K Strack AM Dallman MF Tecott LH Leptin-independent hyperphagia and type 2 diabetes in mice with a mutated serotonin 5-HT2C receptor gene Nat Med 1998 4 1152 1156 10.1038/2647 9771748

[B17] Melcescu E Griswold M Xiang L Belk S Montgomery D Bray M Del Ben KS Uwaifo GI Marshall GD Koch CA Prevalence and cardiometabolic associations of the glucocorticoid receptor gene polymorphisms N363S and BclI in obese and non-obese black and white Mississippians Hormones (Athens) 2012 11 166 177 10.14310/horm.2002.1344 22801563

[B18] Lam SP Fong SY Ho CK Yu MW Wing YK Parasomnia among psychiatric outpatients: a clinical, epidemiologic, cross-sectional study J Clin Psychiatry 2008 69 1374 1382 10.4088/JCP.v69n0904 19193338

[B19] Uwaifo GI Koch CA, Chrousos GP Obesity-associated hypertension Contemporary Endocrinology Endocrine Hypertension 2013 New York: Springer 251 288

